# Supplementary material for: An assessment of the evidence for antibacterial activity of stinging nettle (Urtica dioica) extracts
Source: Access Microbiol. 2022 Mar 24;4(3):000336. doi: 10.1099/acmi.0.000336 (PMC9175978; doi:10.1099/acmi.0.000336)
Supplement: Supplementary material 1 [file acmi-4-0336-s001.pdf]

Supplementary Figures for

An assessment of the evidence for antibacterial activity of stinging nettle (*Urtica dioica*) extracts

Freya Harrison[1], Jessica Furner-Pardoe[1,2], Erin Connelly[1]

[1]School of Life Sciences, Gibbet Hill Campus, University of Warwick, Coventry CV4 7AL, UK
[2]Warwick Medical School, Gibbet Hill Campus, University of Warwick, Coventry CV4 7AL, UK

Corresponding authors: FH f.harrison@warwick.ac.uk & EC erin.connelly@warwick.ac.uk

This file contains Figures S1 and S2

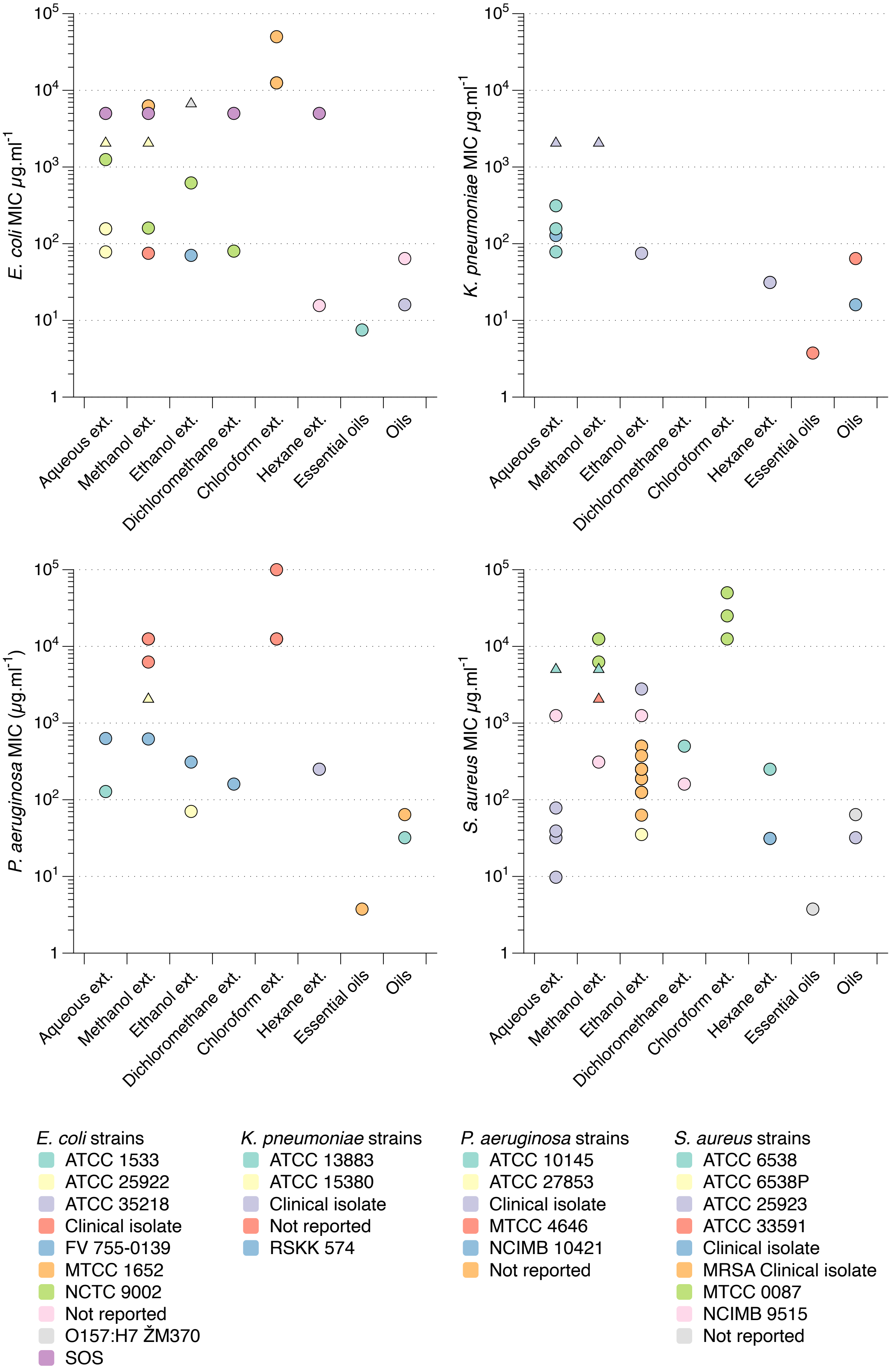

Figure S1. Data from Figure 1, colour coded by bacterial strain or isolate tested.

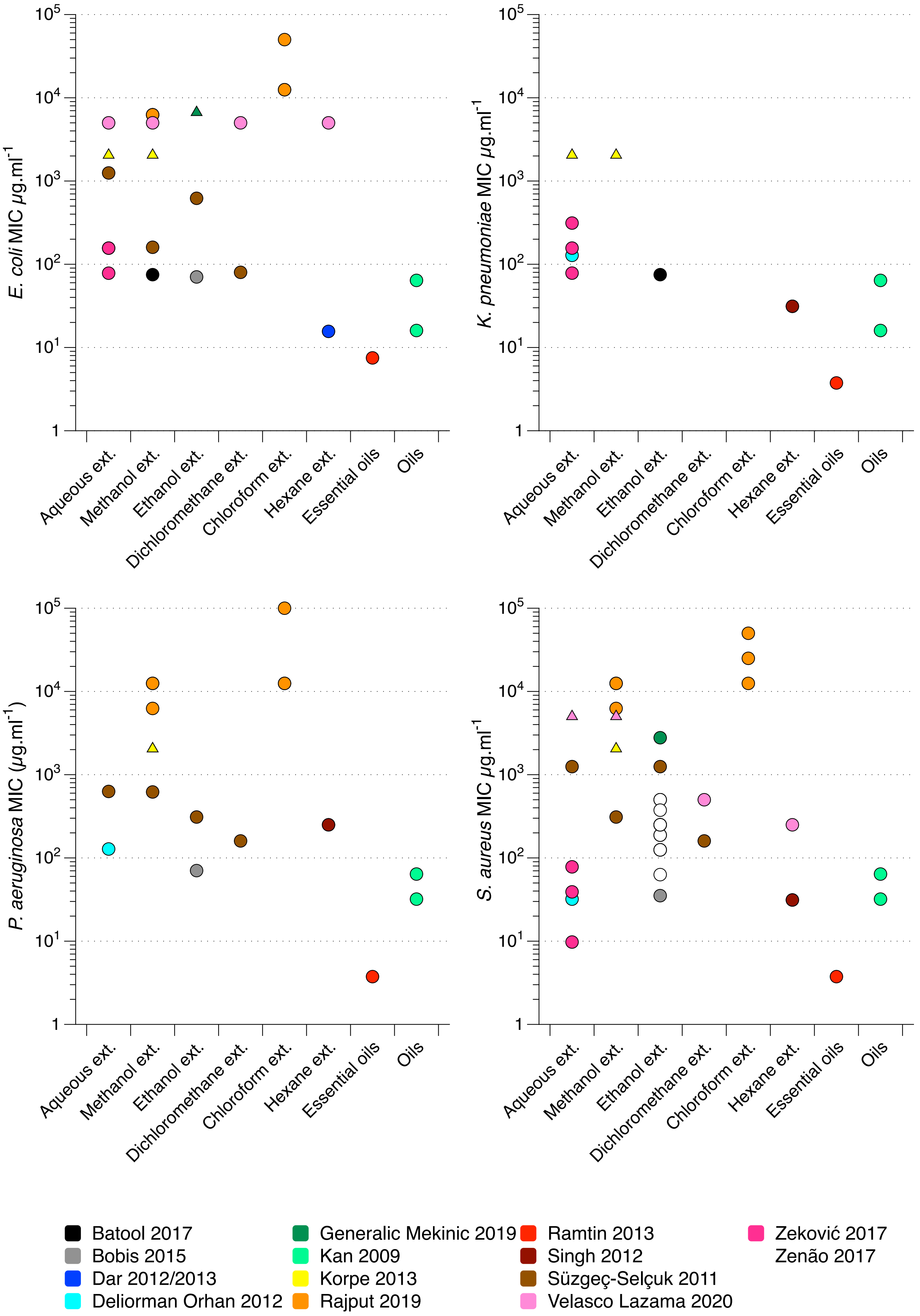
E. coli MIC μg.ml-1
K. pneumoniae MIC μg.ml-1
P. aeruginosa MIC (μg.ml-1)
S. aureus MIC μg.ml-1
Aqueous ext.
Methanol ext.
Ethanol ext.
Dichloromethane ext.
Chloroform ext.
Hexane ext.
Essential oils
Oils
Batool 2017
Bobis 2015
Dar 2012/2013
Deliorman Orhan 2012
Generalic Mekinic 2019
Kan 2009
Korpe 2013
Rajput 2019
Ramtin 2013
Singh 2012
Süzgeç-Selçuk 2011
Velasco Lazama 2020
Zeković 2017
Zenão 2017

Figure S2. Data from Figure 1, colour coded by the study from which the data point was extracted.
